# Torsade de Pointes Triggered by Early Ventricular Escape Beats in a Patient with Complete Atrioventricular Block

**DOI:** 10.1155/2016/7919642

**Published:** 2016-04-06

**Authors:** Erkan Yildirim, Baris Bugan, Suat Gormel, Uygar Cagdas Yuksel, Murat Celik, Yalcin Gokoglan, Serdar Firtina, Sinan Iscen, Emre Yalcinkaya, Ugur Kucuk, Hasan Kutsi Kabul

**Affiliations:** ^1^Department of Cardiology, Gulhane Military Medical Academy, 06010 Ankara, Turkey; ^2^Cardiology Service, Girne Military Hospital, 99300 Girne, Northern Cyprus, Mersin 10, Turkey

## Abstract

Torsade de pointes is an uncommon and malignant form of polymorphic ventricular tachycardia and associated with a prolonged QT interval, which may be congenital or acquired. Complete atrioventricular block may cause QT interval prolongation and torsade de pointes. In this paper, we present a case with complete atrioventricular block complicated with frequent episodes of torsade de pointes triggered by early premature ventricular contractions despite normal QT intervals.

## 1. Introduction

Syncope is a frequent symptom in patients with complete atrioventricular block (CAVB) [1]. In the majority of cases, low cardiac output caused by decreased heart rate is responsible for the symptoms. However in some cases bradycardia associated QT prolongation may lead to malign ventricular tachyarrhythmias causing syncope [1, 2]. We, herein, reported a case with CAVB complicated with frequent episodes of torsade de pointes (TdP) triggered by early premature ventricular contractions (PVCs) despite normal QT intervals.

## 2. Case Report

**Figure 1:**
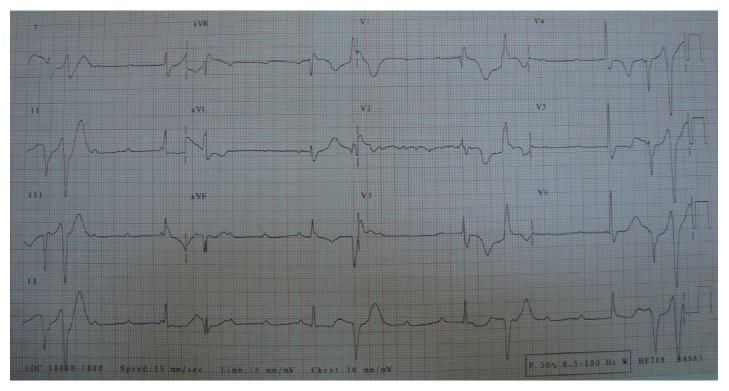
ECG showed CAVB with frequent PVCs. ECG: electrocardiogram, PVCs: premature ventricular contractions.

**Figure 2:**
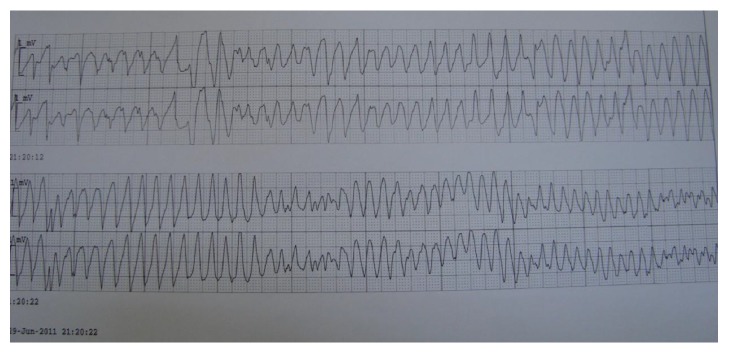
Bedside monitor showed TdP. TdP: torsade de pointes.

**Figure 3:**
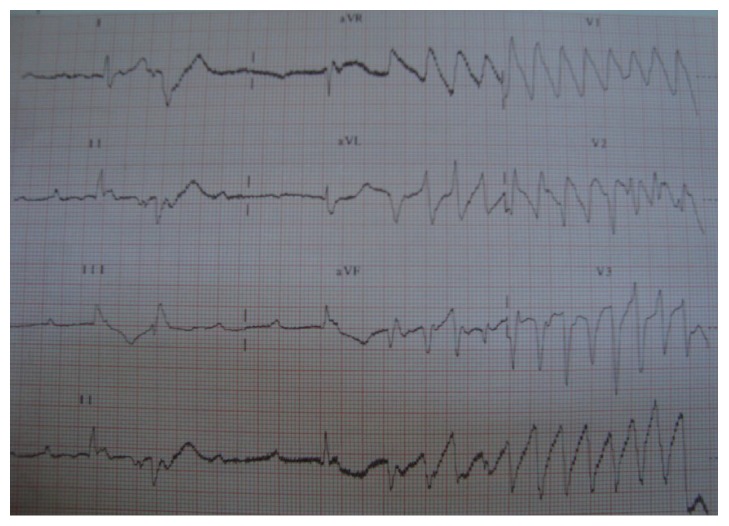
A TdP episode triggered by an early PVC which hit the descending portion of the T wave on ECG. ECG: electrocardiogram, TdP: torsade de pointes.

A 73-year-old woman was admitted to our clinic for fainting and syncope. She had several episodes of syncope in the last few hours. She was on oral antidiabetics for diabetes mellitus and ramipril for hypertension. Her past medical history was otherwise normal. On initial physical examination, she was bradycardic (36 bpm), but alert and fully oriented. Her blood pressure was 100/70 mmHg. The physical examination was otherwise normal. Her initial electrocardiogram (ECG) showed CAVB with a rate of 38 bpm and with frequent PVCs (Figure 1). Her blood chemistry was normal. Bedside echocardiogram revealed no structural heart disease. While being monitored in the coronary intensive care unit she suddenly developed TdP leading to syncope which required DC cardioversion for termination (Figure 2). She had several other episodes of sustained and nonsustained TdP until transvenous temporary pacemaker was in effect. All TdP episodes were triggered by an early PVC which hit the descending portion of the T wave on ECG (Figure 3). Increasing the heart rate by temporary cardiac pacing completely abolished the PVCs and TdP episodes. Coronary angiogram showed noncritical atherosclerotic plaques and ventricular tachycardia (VT) was not induced with electrophysiology study. The patient was further treated with a dual permanent pacemaker. The 6-month follow-up was uneventful.

## 3. Discussion

The term TdP refers to a VT characterized by QRS complexes of changing amplitude that appear to twist around the isoelectric line and occur at rates of 200 to 250/min. TdP during bradyarrhythmias has been reported to be associated with gender, degree of QT prolongation and duration of bradyarrhythmia. Between 5% and 30% of patients with CAVB have been reported to develop TdP [1]. Although many predisposing factors have been cited for TdP, the most common causes are congenital syndromes, severe bradycardia, potassium depletion, and use of medication such as class IA, IC, or III antiarrhythmic drugs [1, 2].

In patients with bradycardia-induced TdP, a number of ECG parameters during bradycardia are correlated with increased risk of TdP including QT interval, T wave morphology, and T peak to T end (Tp-Te) [2, 3]. Although ECG parameters can be reasonable predictors of TdP in bradyarrhythmias, there are limited data on cellular or genetic mechanisms of bradycardia-induced TdP [4, 5].

CAVB may lead to downregulation of potassium channels, QT interval prolongation, and TdP [6]. However, QTc and serum potassium levels were within normal limits in this case. Genetic factors and female gender might have an important role as risk factors for this patient. In addition, electrocardiogram showed us VPCs in which R waves superimposed on T waves as “R-on-T” phenomenon (Figure 3). We believed that these early beats were triggering TdP. The “R-on-T” phenomenon was first described by Smirk in 1949. He described the early beats as precursors for ventricular fibrillation and sudden death [7].

Fries et al. [8] reported the incidence of “R-on-T” phenomenon in patients with implantable cardioverter defibrillators. The study showed that 15% of VPCs initiated VT. Of the VPCs that led to VT, 16% occurred on the ascending limb, 23% on top, and 61% on the descending limb of the T wave. VPCs leading to TdP were more likely to occur on the descending limb of the preceding T wave as seen in our case (Figure 3) [8].

In conclusion, acquired CAVB may sometimes induce TdP and the episodes of TdP result in syncope, cardiac arrest, and even death due to degeneration into ventricular fibrillation. PVCs especially “R-on-T” phenomenon should alert physicians as precursors for ventricular fibrillation and sudden death. Early recognition and implantation of cardiac pacemaker can be lifesaving. Meanwhile all attempts should be made for findings of any underlying causes.
